# Contrasting the effects of intra-uterine smoking and one-carbon micronutrient exposures on offspring DNA methylation

**DOI:** 10.2217/epi-2016-0135

**Published:** 2017-02-17

**Authors:** Rebecca C Richmond, Bonnie R Joubert

**Affiliations:** 1 MRC Integrative Epidemiology Unit, School of Social & Community Medicine, University of Bristol, BS8 2BN, UK; 2 National Institute of Environmental Health Sciences, Research Triangle Park, NC 27709, USA

**Keywords:** DNA methylation, epigenetic epidemiology, epigenome-wide association study (EWAS), folate, maternal smoking, micronutrients, one-carbon metabolism

## Abstract

Maternal smoking and micronutrient intake during pregnancy are two strong biological candidates for impacting the developing epigenome. The extent to which DNA methylation in offspring is modified by these intrauterine exposures has not been presented in parallel. In this review, we summarize human studies which have investigated genome-wide DNA methylation in the offspring in relation to maternal smoking and one-carbon micronutrient exposure during pregnancy. We contrast the primarily independent efforts for these two categories of exposure, and potential explanations for these differences. We emphasize methodological considerations such as power to detect methylation signals, exposure assessment, control of sources of variability, causal inference and the role of observed methylation changes in mediating downstream outcomes in the offspring.

DNA methylation profiling is the most widely used technique to evaluate the impact of early life exposures on the newborn’s epigenome. With the advent of the Illumina Infinium Methyl450 Beadchip (450K) [1], researchers can evaluate CpG-specific DNA methylation differences at sites spread across the epigenome in large epidemiological studies. After the initial flood of research to apply this technology in epigenetic epidemiology, with a focus on performing epigenome-wide association studies (EWAS), the dust has begun to settle on the field for some important exposures considered in human studies.

We review two categories of exposures that have been considered strong biological candidates for impacting the developing epigenome: maternal smoking during pregnancy and maternal one-carbon micronutrient exposures during pregnancy. The extent to which DNA methylation in offspring is modified by maternal periconceptional micronutrients compared with smoking exposures has not been presented in parallel. In this review, we focus on human studies measuring these exposures for the mother during pregnancy and the genome-wide CpG-specific DNA methylation in newborns or children of these mothers (related to exposure). We contrast the primarily independent epigenomic efforts for these two exposures, address research needs and suggest potential future directions.

## Exposures of interest

### Maternal smoking

Cigarette smoke, as well as being a known carcinogen which has detrimental health consequences for smokers, is a well-established toxicant associated with many health effects in the offspring of those exposed *in utero*, including both adverse birth outcomes and later life health outcomes [2]. Smoke exposure has a profound effect on epigenetic profiles and genome-wide DNA methylation changes have been identified in response to both personal smoking and perinatal exposure [3]. Alterations in DNA methylation are one possible mechanism mediating the harmful effects of smoke exposure, and both candidate gene [4] and EWAS [5] have identified methylation changes in gene regions involved in the etiology of smoking-relating outcomes. In many respects, the investigation of methylation changes related to prenatal smoke exposure represents a ‘flagship’ exposition of epigenome-wide approaches for investigating maternal exposures, with an increasing number of methylation signatures in regions of the genome being consistently replicated between studies.

### Maternal one-carbon micronutrients

Micronutrients are essential nutrients that play a crucial role in fetal development, most notably by the prevention of neural tube defects in newborns by maternal folic acid supplementation before and early in pregnancy. Severe deficiencies in micronutrients such as folate, iron, zinc and various vitamins can lead to adverse pregnancy outcomes, particularly in undernourished populations [6]. Micronutrients particularly relevant to epigenetic mechanisms include those involved in the one-carbon metabolism pathway [7], including folate, choline, betaine and other B vitamins. The one-carbon metabolism pathway provides methyl groups for a range of biochemical reactions including methylation of DNA which impacts gene expression. Differences in genome-wide DNA methylation in newborns has been evaluated in relation to maternal folate and other micronutrient exposures in candidate gene methylation studies [8] and EWAS [5], where folate is the predominant nutrient showing significant associations with the developing epigenome. Comparison of effects in nourished versus undernourished populations remains to be fully elucidated as well as the extent to which single micronutrients or combinations of micronutrients impact methylation differences. Given the relevance to the epigenome, we reviewed the research evaluating maternal one-carbon micronutrient exposures in pregnancy and epigenome-wide DNA methylation in newborns and children.

## Literature review

We searched the literature (PubMed, Scopus and Web of Science databases) in September 2016, to identify novel publications describing maternal smoking or micronutrient exposures during pregnancy and the offspring epigenome. The micronutrients included in our search were those identified to play important roles in one-carbon metabolism [7]: Folate, choline, betaine, methionine, vitamin B2 (riboflavin), B6, B12 (cobalamin) and homocysteine. Details of the search strategy are included in the Supplementary Material.

### Epigenetic effects of maternal smoking

Single-site and global methylation associated with maternal smoke exposure has been previously evaluated [4]. In that review, the authors highlighted then current data suggesting that maternal smoking influences many different regions of the epigenome, and thus emphasized the importance of interrogating associations on an epigenome-wide scale. Therefore, we focused our search on those studies which have used genome-wide methylation arrays to compare methylation profiles from offspring of women who reportedly smoked during pregnancy with those of offspring whose mothers did not smoke.

Of the 24 studies which met our search criteria (Supplementary Table 1); [5,9–31], publication dates ranged from 2011 to 2016. The most common platform which has been used is the Illumina Infinium HumanMethylation450 (450K) followed by the Infinium HumanMethylation27 (27K) Beadchip, used typically in earlier studies. One study investigated promoter-based CpG sites using the Affymetrix Human Promoter 1.0R array [26] and another investigated whole-genome bisulfite sequencing [10]. Sample sizes for the 24 studies varied from just nine mother–offspring pairs [31] to 6685 mother–offspring pairs in a meta-analysis across 13 cohorts [5]. Measures of smoke exposure varied from retrospective report, prospective report and objective measures of cotinine, with a few studies using a combination of cotinine and self-report [9–10,14–16,23]. The most common tissue in which methylation was investigated was cord blood, followed by peripheral blood at later ages, although placenta and lung tissue were also investigated [12,20,28], with some studies investigating more than one tissue [5,12–13,19,24].

Consistent with this, the most common time point at which methylation was measured was at the birth of the offspring, while other studies assessed methylation post conceptually [12], in infancy [10,30], childhood [5,10–11,13,18–19,24–25], adolescence/early adulthood [9,13,19,24,32] as well as into adulthood [27]. Some studies investigated methylation at multiple time points longitudinally [10,13,19,24].

In terms of the methods applied, the majority of studies performed EWAS using multivariable linear regression models with adjustment for potential confounding factors (most commonly maternal age, offspring sex, socio-economic position, paternal/postnatal smoking) as well as cell count adjustment, generally using a reference-based approach [33], and various technical covariates. Some studies investigated the impact of differing exposure assessment, for example, comparing cotinine measures with self-reported questionnaire data [15] or assessing differences between any reported smoking in pregnancy versus sustained smoking throughout [5]. Furthermore, some studies were able to compare associations between maternal smoke exposure during pregnancy with paternal [16–17,24] and grandparental smoke exposure [16], as well as postnatal smoking effects [5,11,18–19,24–25].

With respect to replication and validation of findings, the majority of studies highlighted overlap between their findings and other results from the literature with a few studies performing formal replication of top sites in independent datasets [11,15,17,19,21]. In addition, validation of differential methylation at CpG sites was assessed in a few studies with the use of other technologies such as pyrosequencing [10,12,20,26,28–29].

While the majority of these previous studies have used an EWAS design, focusing on methylation differences at individual CpG sites across the genome, some studies have taken alternative approaches such as the assessment of differentially methylated regions [10,27], gene sets [26] and use of Bayesian Mixture Modeling [24] for dimensionality reduction. Furthermore, a couple of studies have used methods to better capture latent confounding and identify additional signals [30,32]. Other work developed a methylation prediction score from a maternal smoking EWAS to classify maternal smoking status in a test dataset [23].

**Figure 1.**
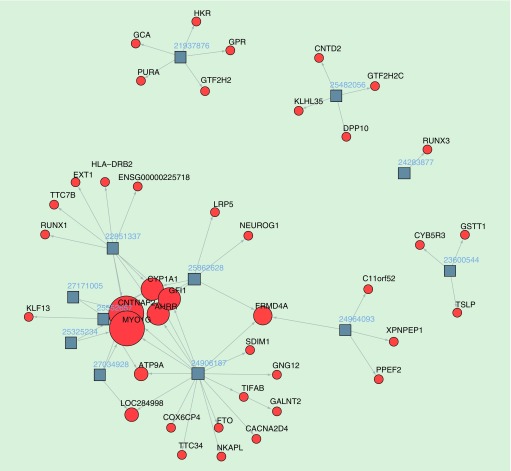
**Network visualization of individual studies investigating maternal smoking in relation to offspring DNA methylation.** Squares represent individual studies; numbers represent related PubMed IDs; circles represent gene regions; text represents annotated gene names; arrows represent the links between the studies and the gene regions, in other words, highlighting studies in which CpG sites have been identified in these gene regions at either Bonferroni significance or with replication/validation attempts; the size of the circle is proportional to the number of independent studies in which the gene regions have been identified. N.B. this network plot does not include the recent epigenome-wide association studies meta-analysis for maternal smoking (PMID 27040690).

An amassing number of EWAS for maternal smoking has led to the identification of an abundance of strong, highly replicated methylation signatures. Figure 1 shows a network plot of the top gene regions identified in individual studies in relation to maternal smoking status. In particular, methylation at CpGs in *AHRR*, *CYP1A1*, *MYO1G*, *CNTNAP2*, *GFI1* and *FRMD4A* has been consistently implicated in relation to prenatal smoke exposure, most commonly in those studies investigating DNA methylation in cord blood. However, overlap with some studies assessing methylation in peripheral blood into childhood and adolescence is evident, implying a lasting effect of maternal smoking in pregnancy on offspring DNA methylation profiles [5,24].

These gene regions have been implicated in pathways related both to regulation of biological processes to the exposure (e.g., *AHRR* and *CYP1A1*, which are both involved in the detoxification of tobacco smoke [34]) as well as possible consequential processes of the prenatal smoke exposure (e.g., *FRMD4A* which has been associated with nicotine dependence [35] and *MYO1G*, *CNTNAP2* and *GFI1* which are involved in various developmental processes [19,24–25,36–37]).

CpG sites at *MYO1G*, *CYP1A1* and *FRMDA4* typically show an increase in cord-blood methylation in relation to maternal smoking whereas *AHRR*, *CNTNAP2* and *GFI1* show a decrease. This is reflective of changes across the whole genome, where smoking during pregnancy has been associated approximately equally with hyper and hypomethylation [5]. The average percent change in methylation at these sites has shown to be small, ranging from -8 to 7% in the study by Markunas *et al.* [21]. Further annotation of the CpG sites in these gene regions illustrated a significant enrichment for CpG shores/islands and intronic/intergenic locations [21].

In terms of the impact of methylation changes on gene expression, this has been most consistently investigated in relation to *AHRR*. Cg05575921, the site most strongly associated with both maternal smoke exposure and own smoking [3] has been shown to be inversely associated with *AHRR* expression in different tissues [38]. Upregulation of *AHRR* expression acts as a negative regulator of the aryl-hydrocarbon receptor (AhR) pathway, and in turn suppresses *CYP1A1* transcription, another gene strongly implicated in relation to maternal smoking. Consistent with this, smoking has an opposite impact on DNA methylation at sites in *CYP1A1* (hypermethylation) to *AHRR* (hypomethylation) in cord blood, which is anticipated given the opposing function of these genes in the AhR pathway. Interestingly, however, *CYP1A1* has been found to be hypomethylated in placenta, which is correlated with increased expression in this tissue [39].

The proposed biological implications of these gene expression changes are also conflicting, with some studies suggesting that the inhibition of the AhR pathway may compromise the body’s capacity to metabolize harmful environmental chemicals among those exposed to smoke [3], whereas others suggest that the feedback response of *AHRR* in relation to smoke exposure is actually adaptive because it inhibits the released of carcinogenic metabolites produced by the AhR pathway [40], which is in part corroborated by the role of AHRR as a putative tumor suppressor [41]. The impact of smoke-induced methylation changes in relation to cancer outcomes are starting to be evaluated [42] and may have potential relevance in this context for implicating methylation in the causal pathway between maternal smoke exposure and increased risk of childhood cancers [43]. Furthermore, the downstream consequences of methylation changes at other CpG sites associated with maternal smoking in relation to some perinatal and childhood outcomes have also been investigated in a mediation context [10,20,23,28–29] although the causal relevance of these findings require further evaluation (see section 'Mediation').

### Epigenetic effects of maternal one-carbon micronutrients

Research on the influence of micronutrients on DNA methylation falls within a larger field of nutritional epigenomics. Maternal nutrition impacts fetal development and there is some evidence of modifications to the newborn epigenome related to maternal micronutrient exposures in pregnancy. However, despite a topic of interest for many years, the data demonstrating newborn epigenetic effects of maternal micronutrients during pregnancy is substantially less than the evidence for the effects of maternal smoking on the offspring epigenome.

Of the seven studies which met our search criteria (Supplementary Table 2) [44–50], publication dates ranged from 2012 to 2016. The most common platform which has been used is the 27K (four studies) followed by the 450K (three studies). All seven micronutrient studies focused on maternal folate during pregnancy. Although one study [48] evaluated other nutrients such as betaine, choline and other nutrients involved in one-carbon metabolism, the strongest effects on offspring methylation were observed for maternal folate levels in pregnancy. Sample sizes for the seven studies ranged from 18 neonates of mothers enrolled in a clinical trial [46] to nearly 2000 mother-offspring pairs in a meta-analysis of two independent European cohorts [48]. Measures of folate exposure included measurements from maternal serum, plasma or blood samples as well as maternal report of folic acid supplementation. All studies measured methylation in cord blood samples, while one study also measured methylation in peripheral blood in infancy [45,49]. Only one study [49] evaluated methylation at multiple time points (birth and 9 months) and observed a stronger effect at the later time point.

The statistical methods applied in the micronutrient studies included the epigenome-wide association analysis using multivariable linear regression models with adjustment for potential confounding factors, but many studies were underpowered for this approach. The earliest publication used Illumina’s methylation module for data analysis of 27K data [49], whereas other studies using clustering approaches such as principal components analysis [44] or hierarchical clustering [46]. Studies published in 2015 and 2016 accounted for cell type as a covariate in statistical models whereas earlier studies did not necessarily consider cell type heterogeneity. One study [45] measured the *MTHFR* genotype and incorporated a Mendelian randomization approach to data analysis.

**Figure 2.**
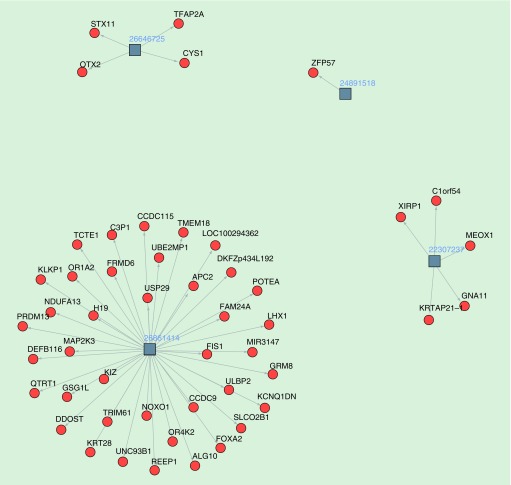
**Network visualization of individual studies investigating maternal folate in relation to offspring DNA methylation.** Squares represent individual studies; numbers represent related PubMed IDs; circles represent gene regions; text represents annotated gene names; arrows represent the links between the studies and the gene regions, in other words, highlighting studies in which CpG sites have been identified in these gene regions at either Bonferroni significance or with replication/validation attempts; the size of the circle is proportional to the number of independent studies in which the gene regions have been identified.

Regarding replication, only one study evaluated more than one population, in this case with meta-analysis rather than independent replication analysis [48]. Other studies evaluated one study population. Figure 2 shows the comparison of results across studies. Not surprisingly, there is minimal replication of findings across the micronutrient studies. This likely reflects many things: the differences in sample type, study design, study population, microarray coverage and statistical modeling.

## Comparison of EWAS findings

Stronger statistical significance (in terms of the number of EWAS-significant sites) was observed for the maternal smoking studies compared with the micronutrient studies, and methylation sites were more consistently replicated between studies. Comparing the largest studies for both maternal smoking and maternal folate to date [5,48], the manuscript evaluating maternal folate in pregnancy included only two cohorts with an overall sample size of 1988 newborns, whereas the paper evaluating maternal smoking during pregnancy evaluated 13 independent cohorts with an overall sample size of 6685 newborns. The smoking study reported 6073 CpGs reaching false discovery rate (FDR)-corrected statistical significance whereas the folate study observed 443 FDR significant CpGs.

Although there is a substantial difference in sample size, maternal smoking appears to have a more far-reaching impact on the methylome in terms of the number of epigenome-wide significant sites which have been consistently replicated between studies (Figures 1 & 2). It is possible that folate impacts just a few important genes or pathways, whereas smoking has more multifaceted biological implications reflected in the number of pathways and genes identified. However, outside of considering true impact of the compared exposures on the epigenome, there are several statistical factors that may explain the discrepancy between the robustness of EWAS hits and minimal overlap of findings for maternal micronutrient exposures compared with the maternal smoking publications.

## Statistical methodology

The statistical challenges of EWAS have been discussed in detail elsewhere [51–54], and are also relevant for the investigation of both maternal smoking and micronutrient exposure. For example, low statistical power, publication bias, noise in the exposure variable, as well as various confounding factors threaten the detection of biological signals of interest and the ability to infer causality from to the epigenomic modifications associated with maternal smoke and micronutrient exposures.

Methods for improving the robustness of EWAS findings have been implemented in some of these existing studies, predominantly in relation to maternal smoking, although areas for improvement include: further investigation into the magnitude and persistence of effects on methylation; improving power for detecting methylation signals; careful exposure assessment; accounting for cell type heterogeneity; and assessing causality with respect to both the maternal exposure and in relation to the observed methylation changes in mediating downstream outcomes which have been implicated.

### Persistence of effects

Persistence in a methylation signal over time can provide insight into epigenetic effects of early life environmental exposures. For example, prospective studies investigating associations between maternal smoking in pregnancy and peripheral blood methylation in offspring during childhood and adolescence [5,24] have identified strong similarity with smoking-associated methylation in newborns, implying a lasting effect of maternal smoking in pregnancy on offspring DNA methylation profiles at these sites. However, one paper from the micronutrients review evaluated more than one time point in an individual which found a stronger effect of exposure slightly later in life rather than early and persistent effects [49]. In contrast, an independent study published subsequent to conducting our literature search measured methylation in cord blood at birth, whole blood at age 7, and substance use in adolescence at ages 14–18 and found that methylation differences at birth predicted earlier initiation and longer term substance abuse in adolescent life better than methylation differences at age 7 [55]. Furthermore, these methylation differences were suggested to partially mediate the effect of prenatal maternal smoke exposure on adolescent substance use (see section 'Mediation').

These contrasting findings suggest that additional research is warranted to clearly differentiate the independent and combined effects of environmental exposures over time on later life DNA methylation and health end points. It is also likely that these patterns differ across assessed environmental exposures.

### Assessing power for detecting methylation signals

Figures 1 & 2 highlight some of the isolated maternal smoking and one-carbon micronutrient studies where findings have not been consistently replicated. These are typically those studies with small sample size where methylation differences have been validated with the use of pyrosequencing, but without replication in an independent study population. While it is possible that these signals represent false positive findings, the results of a larger meta-analysis not included in this figure and described below [48] identified some sites which were not previously replicated in the individual studies from Figure 1. This indicates that some of these sites, while not showing replication in previous studies, may indeed be true positives. Another potential explanation for sites which have not been replicated is that these methylation signals are an artifact of methylation platform (27K vs 450K), or alternatively the result of methylation profiling in different tissues (e.g., placenta) or populations (e.g., ethnic groups), although these studies were performed in samples from individuals of predominantly European ancestry. These factors may also explain the lack of replication of CpG sites in relation to maternal micronutrient status in Figure 2.

To improve statistical power which will be more equipped to account for noise in the exposure, larger sample sizes are needed in studies as well as replication. Meta-analysis is the most conventional means of improving power in GWAS, which has recently been adopted by the epigenetics field, for example by the Pregnancy and Child Epigenomics (PACE) consortium, following models from GWAS consortia such as Cohorts for Heart and Aging Research in Genomic Epidemiology (CHARGE) and Early Growth Genetics (EGG).

The PACE consortium has meta-analyzed, across 13 cohorts and 6685 individuals, the association between maternal smoking in pregnancy and DNA methylation in newborn cord blood [5]. In this study, over 6000 CpG sites were found to be differentially methylated, including sites previously identified in smaller EWAS studies (Figure 1) as well as many novel loci. Furthermore, evidence for persistence of methylation marks at older ages in the offspring exposed to prenatal smoke exposure has been corroborated in this EWAS meta-analysis, where many of the sites identified in cord blood showed differential methylation in five cohorts which also had methylation data available in older children, with 73% of sites showing a consistent direction of effect and 148 CpGs identified with FDR significance at replication level [5].

Furthermore, this meta-analysis identified differential methylation in several genes which were also found to be enriched in development and disease pathways, including genes relevant to outcomes associated with smoke exposure such as orofacial clefts, asthma and certain cancers. The study also evaluated the relationship between significant CpGs and gene expression in two independent study populations. However, the study did not investigate methylation in any other tissues, which are less accessible in many of the larger cohort studies, and did not formally investigate any of the downstream consequences of the observed methylation changes in terms of phenotypic differences in the offspring, as was attempted in some of the individual studies [10,17,20,23,28–29].

The meta-analysis approach typically requires harmonization of exposure metrics and covariates included in cohort-specific statistical models. For many of the maternal micronutrient studies (Supplementary Table 2), we observed substantial variability in these features. Thus, a meta-analysis or pooled analysis approach may be most successful if collaborations discuss design strategies before implementing procedures (e.g., synthesizing methods for assessing micronutrients, DNA methylation pre-processing and quality control, time points of sample collection, etc.), while maintaining unique study population characteristics.

### Exposure assessment

A major challenge in epidemiological studies is to ensure adequate exposure assessment. This includes not only measuring exposure accurately but also accounting for the timing and dose of the exposure. There are several ways in which the smoking and micronutrient exposures contrasted here differ in how they are assessed and what biases may impact results. For most study populations, there is stigma related to smoking during pregnancy, which increases the likelihood of underreporting true smoking exposure by the mother. To address this, some studies use cotinine, a biomarker for smoking, to objectively capture exposure at a single point in time. Although a single measure of cotinine at one point in pregnancy will not adequately capture all exposure throughout pregnancy, it can provide insight into how accurately the self-reported data correlates to biomarkers of exposure.

For micronutrient exposures during pregnancy, there may be aspects of both under and over reporting of exposure, depending on dietary information collected, supplement use and the presence of biomarker metrics. Given the variability in how reports may be biased and generally noisy, it may be even more crucial to obtain biological specimens from the mother during pregnancy such as plasma, serum and blood, and to obtain the most stable biomarkers of exposure such as red blood cell folate rather than serum or plasma-based measurements. Validation of micronutrient exposure may also be more complicated than it is for smoking as there are multiple routes of exposure (diet, supplement use, co-exposures that influence metabolism) and multiple ways of measuring exposure (biomarkers, food frequency questionnaire data, study-specific questionnaires). As such, it is crucial for nutritionists to be involved in this complicated exposure assessment in addition to the standard molecular epidemiology and statistical team.

Differences in the extent and robustness of methylation signals may reflect the route of exposure. For example, the associations found between maternal cotinine levels, an objective biomarker of smoking and DNA methylation in newborns implies a dose-dependent effect of maternal smoking in pregnancy [15]. This dose-response reflects the direct inhalation of tobacco smoke, whereas micronutrient exposures are primarily dietary through food or supplements. It is possible the interaction with other foods, the consistency of intake, or the role of metabolism are involved in the ultimate dose of micronutrient exposure within the body. In many ways the micronutrient exposures are substantially noisier and with greater variability within a person at a given point in time, over time and within a study population. This variability will reduce power to detect statistically significant associations after applying correction for multiple testing.

Another consideration in exposure assessment is timing of measurement. With respect to the literature on maternal smoking EWAS, several studies have highlighted the importance of sustained smoking during pregnancy [5,16,24], rather than smoking around the periconceptional period, which has been found to be independent of smoking intensity [16]. This is interesting given *a priori* knowledge that epigenetic profiles are typically established in early development and propagated during embryogenesis [56]. Rather, it suggests that cumulative exposure to environmental stimuli *in utero* might have a greater impact on the developing offspring than restricted exposure windows. We did not find many longitudinal studies evaluating exposure at multiple windows in this review, but recommend this for future research. This is particularly of interest for epigenomic studies of maternal micronutrients, given the preferential timing of supplementation during the periconceptional period [57].

Another notable weakness in both the smoking and micronutrient studies published to date is the underrepresentation of minority study populations, who may have variable exposure and related characteristics compared with study populations primarily of European ancestry (Supplementary Tables 1 & 2).

Given robustness of methylation marks in relation to an exposure of interest, one strategy is to use epigenetic signatures to estimate the existence and magnitude of exposure by serving as prediction markers. A recent study developed a methylation prediction score from a maternal smoking EWAS to classify maternal smoking status in an independent dataset [23]. Such a score may serve as an archive of historical exposure, for example, when no smoking history has been collected in a study, and as a means of assessing exposure misclassification, for example, to assess misreporting of smoking behavior during pregnancy or to substitute for poorly measured dietary data.

### Assessing causality

Findings from EWAS of maternal exposures such as smoking and micronutrient are of particular utility for investigating hypotheses on the proposed epigenetic processes underpinning intrauterine effects on offspring health and development [58]. However, it is important to realize that methylation signatures are essentially phenotypic, and are therefore subject to the same potential problems of confounding, reverse causation and bias which afflict observational epidemiology [54,59].

While, in the context of prenatal exposures, reverse causation is not a major concern given it is unlikely that the offspring’s methylome will directly influence the maternal phenotype, the potential for bias and confounding threaten the detection of true causal effects. Bias has been exemplified with the poor replication of some methylation sites between different EWAS (Figures 1 & 2), given the extent of multiple testing and increasing probability of false positive findings in this context. As mentioned, key strategies to reducing the likelihood of such bias are through increasing power in individual studies and through meta-analysis.

Furthermore, it is possible that the associations observed between prenatal exposures and offspring DNA methylation are subject to confounding, due to the potential presence of factors which modify methylation profiles and are also associated with the exposures.

### Cell type heterogeneity

One major factor thought to lead to spurious results in EWAS is ‘confounding’ by cell-type heterogeneity. Although this is not confounding in the strict sense, since cell type proportions of the samples being profiled for methylation are unlikely to influence the maternal exposure, if cellular differences are found to underlie the observed methylation changes this has consequences in terms of the interpretability of the EWAS findings [60] or in CpG-specific findings [61,62]. It is therefore important to correct for cell type in the setting of maternal exposures such as smoking and micronutrients, which have been found to influence cell type proportions, with the use of reference-based [33,63] or reference-free approaches [64]. In the meta-analysis of maternal smoking by Joubert *et al.*, a method of cell type correction based on an adult reference panel [33] showed that 78% of sites identified in the main model retained EWAS-significance in cell type-adjusted models.

### Genetic confounding

Interindividual variation in methylation can be a consequence of DNA sequence polymorphisms that result in methylation quantitative trait loci (meQTLs) [65,66]. As such, the role of genetic transmission in explaining an association between a maternal exposure and offspring methylation should ideally be considered when trying to assert causal intra-uterine effects. One simple means of testing this is by ensuring no single nucleotide polymorphisms underlie the methylation probes of interest [67], although long-ranging cis- and trans- effects can explain a great proportion of variability in DNA methylation, indicating the importance of integrating both genetic and epigenetic architecture to delineate effects.

### Residual confounding

The most commonplace method for evaluating and minimizing the impact of confounding in EWAS is with the inclusion of covariates associated with the exposure. In the case of maternal exposures, these potential confounders are most commonly maternal age, parity and socio-economic status. From the EWAS investigated here (Supplementary Tables 1 & 2), various adjustments for potential confounding factors, on the whole, did not substantially attenuate results. However, the ability to adequately account for potential confounders relies on the assumption that they have been identified and measured with little or no error. In the absence of full or detailed information about potential confounders, a matrix decomposition method (such as surrogate variable analysis) may be used to account for unmeasured and residual confounding in an agnostic manner [68].

### Causal inference methods

As well as these conventional strategies to minimize the impact of confounding in EWAS studies, some alternative strategies adapted from observational epidemiological studies may also be used, some of which have been considered in the context of maternal smoking and micronutrient intake and offspring methylation.

The strongest evidence relating maternal exposures to offspring methylation derive from intervention studies, such as those where strong external nutritional factors influence the population under study largely at random and therefore are not typically associated with confounding factors [69,70]. The ‘gold standard’ for causal inference is the randomized controlled trial (RCT), for example, with the randomization of women to different interventions in pregnancy. Such an approach was used in one study [49], where methylation differences were found among the offspring of Gambian women who were enrolled in a placebo-controlled RCT for preconceptional micronutrient supplementation (Supplementary Table 2). Nonetheless, the specificity of the micronutrient exposure in this setting was not confirmed and few trials of individual micronutrients in pregnancy have been reported in relation to methylation changes. In addition, although the gold standard, RCTs are sometimes impossible or unethical to implement, for example, in the context of randomizing women to folic acid supplementation in early pregnancy (for which there are known adverse consequences of folate deficiency on offspring development).

In the absence of RCTs or other intervention designs, various methods have been developed to minimize problems afflicting observational epidemiology and to strengthen causal inference [71], which may be relevant to the epigenetic community and which have already been applied, predominantly in the context of maternal smoke exposure, but increasingly in relation to micronutrient supplementation.

We have already mentioned the utility of examining the strength of association, dose-response and persistence of methylation in response to the maternal exposure. It is also of interest to investigate the specificity of an association. For example, with respect to maternal smoking the finding that methylation in the *AHRR* provides good evidence against confounding given that this gene is directly implicated in the detoxification of tobacco smoke, therefore providing specificity of function. Similarly, for maternal folate some of the CpGs identified in the largest EWAS to date were found to be directly related to folate biology [48].

Nonetheless, the multitude of chemicals in cigarette smoke and complexity of one-carbon metabolism means that these exposures may impact multiple biological pathways, limiting the specificity of the association between this exposure and methylation change in other gene regions. For sites where biological plausibility is less well known, other methods may be used to establish specificity of the association between the maternal exposure and offspring methylation. A negative control approach is one that utilizes an additional exposure or outcome that would be liable to the same sources of confounding, but for which causal associations cannot be plausibly ascribed [72,73]. Any evidence of an association over and above that observed in the negative control is indicative of a causal effect.

A negative control design primarily used for exploring the extent to which associations of intra-uterine exposure might be causally related to offspring outcomes is the parental comparison approach. Such a design has previously been used to establish the causal effect of maternal smoking (and not paternal smoking) on offspring methylation [16–17,24]. In the context of evaluating the long-term effect of prenatal smoke exposure on DNA methylation, maternal smoking after pregnancy may be used as a negative control which would not be expected to have the same effect as smoking during pregnancy if the mechanism of influence is through the intra-uterine environment. In particular, longitudinal studies with repeated measures of smoke exposure both pre- and postnatally are useful at unpicking various life course effects and defining ‘critical periods’ for the establishment of methylation marks [24].

### Mendelian randomization

Mendelian randomization is a method that uses genetic variants robustly associated with modifiable exposures to infer causality [74,75]. As genetic variants can be assumed to be randomly assigned [76], this approach is in principle analogous to an RCT, where study participants are randomly allocated to one or another treatment, avoiding potential confounding between treatment and outcome.

**Figure 3.**
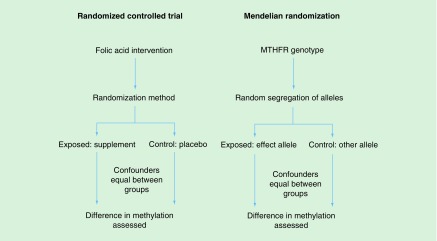
**Comparison of design of a Mendelian randomization study and randomized controlled trial in the context of establishing a causal effect of maternal folate on DNA methylation.**

Mendelian randomization may be used to provide unique insights into the causal nature of intra-uterine exposures, where maternal genotype is taken to be a proxy for environmentally modifiable exposures in pregnancy that influence the intra-uterine environment [77]. For example, genetic variation in *MTHFR* is associated with methylenetetrahydrofolate reductase activity and so with circulating folate levels. As such, genetic variation at *MTHFR* ‘mimics’ the effect of maternal folate supplementation in an RCT (Figure 3). In the context of offspring DNA methylation, the *MTHFR* genotype has been used in a Mendelian randomization context to investigate the causal effect of maternal red blood cell folate on genome-wide methylation in infant cord blood [45].

### Mediation

Given evidence for causal associations between maternal smoking and micronutrient exposures in pregnancy and methylation changes in the offspring, it is important to consider whether induced changes are also associated with perinatal and offspring outcomes attributed to these exposures, and further work is required which may link methylation variation to health and development [58].

With respect to maternal smoking, there is increasing attention on performing downstream analyses, to link methylation differences with transcriptional differences or changes in gene expression [5,10–11,26,28–29]. Furthermore, functional enrichment and pathway analyses have been performed to link the observed methylation differences with biological pathways, highlighting observed enrichment in pathways and processes critical to development and conditions that can be caused by maternal smoking such as orofacial clefts and asthma [5]. Furthermore, some studies have investigated associations between differential methylation and specific offspring outcomes associated with prenatal smoke exposure, including infant weight [23,28–29], gestational age [20], atopic dermatitis [29] and lung function [10]. Similarly, because of the relevance to early life health outcomes such as neural tube defects, some studies such as Price *et al.* [78] have specifically evaluated the relationship between folate levels and neural tube defects to consider mediating effects of methylation.

However, despite these findings it remains to be seen whether the small observed changes in DNA methylation are biologically (e.g., impacting transcription) or clinically relevant and whether the association with offspring phenotypes are truly causal. In particular, whether DNA methylation is a true mediating mechanism of these associations or simply a refined exposure indicator requires further exploration by extending causal inference.

Epidemiological studies have tended to profile methylation signatures from easily accessible sources of DNA, such as cord or whole blood. However, how likely it is that DNA methylation in blood mediates the effect of the *in utero* exposure on a developmental trait, and whether blood cell methylation is representative of the epigenetic state of a target tissue, remains unclear [79]. Tissue specificity therefore limits the assessment of functional consequences of methylation changes. Attempts should be made to investigate concordance of methylation signatures between tissues in relation to a maternal exposure, which can provide more insight into systemic effects, and to investigate mediation of methylation in target tissues more closely linked with the offspring outcomes of interest rather than peripheral tissues [12].

Furthermore, limitations of observational epidemiology (specifically measurement error, confounding and reverse cause) can also afflict conventional mediation approaches and may lead to incorrect conclusions regarding causal effects in the context of methylation change [80]. Solutions to these problems include the use of experimental designs [10] as well as Mendelian randomization [81] which may be used to establish the causal impact of the methylation change on an offspring outcome, independent of the exposure of interest.

**Figure 4.**
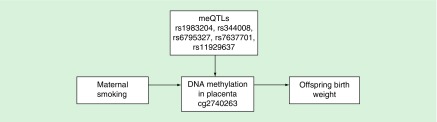
**Mendelian randomization analysis to establish the causal effect of maternal smoking-associated DNA methylation change in relation to offspring birthweight, using genetic variants robustly associated with DNA methylation (meQTLs).** meQTL: Methylation quantitative trait loci.

A study published subsequent to conducting our literature search investigated the role of DNA methylation in mediating the known causal effect of maternal smoking on offspring birthweight using a Mendelian randomization approach [82]. This study was conducted using methylation data from placental tissue, an organ that plays a key role in fetal growth and development, and taking meQTLs robustly associated with changes in methylation at the sites of interest to establish causal effects. Results of the study suggested a causal effect between decreases in placental methylation at a CpG site between *LINC00086* and *LEKR1* and lower birthweight in the offspring (Figure 4). However, the authors of the study state that these results should be taken with caution because of potential pleiotropic effects of the meQTLs on birthweight which cannot be completely ruled out.

## Future perspective

### Implications of findings

In this review, we describe existing literature for two maternal exposures (maternal smoking and maternal micronutrient exposure) in pregnancy and the offspring epigenome. Restricting our review to EWAS only using popular technologies measuring CpG-specific methylation across the genome, we identified 24 relevant maternal smoking studies and seven relevant micronutrient studies. In addition to differences in the underlying biology involved in how these two impact the developing epigenome, there are several methodological considerations to consider, including sample size, combined analyses using meta-analysis, availability of data, challenges to exposure measurement, statistical approaches and assessment of persistent effects. We found the literature describing the influence of maternal smoking in pregnancy on the offspring epigenome to be substantially more populated with regards to the above considerations compared with the micronutrient literature. This may in part be driven by the availability of data across many cohorts and the recent consortium-based efforts for smoking that represent a proof of principle to guide efforts on other environmental exposures. Notably, similar collaborative efforts can be useful to evaluate more ‘noisy’ exposures such as micronutrients given the increase in statistical power and ability to evaluate heterogeneity of effects across studies.

It is possible that maternal smoking during pregnancy has a greater true biological impact on the offspring methylome – impacting more sites and more genes – compared with maternal micronutrient exposures. This makes sense in many ways due to the numerous health effects associated with prenatal and active smoking. In contrast, micronutrients such as folate may represent true biological differences that impact the methylome, but that involve fewer processes, pathways and implicated genes. Further research is warranted to more clearly understand these differences and whether they can be attributed to statistical factors, noise in the data or true biological differences.

### Future directions

With respect to research on maternal micronutrients and offspring methylation, there is a need for more large-scale research in this area, specifically with regard to investigating the independent and combined effects of micronutrients other than folate. To understand the broader reaching impact of micronutrients on the human methylome, efforts to further replicate findings using independent epidemiological study populations is needed. Publications demonstrating the lack of replication are also important to avoid unnecessary repeated efforts, although this is a tricky argument due to population and methodological differences across studies.

Ongoing efforts with meta-analysis or pooled analyses is very important. This not only increases statistical power to detect effects but can offer many insights into how exposure and effects vary across independent study populations. In order for this to be successful in relation to maternal micronutrients, harmonization of exposures in different studies first needs to be established. Furthermore, metrics used in GWAS such as p-values for heterogeneity can be evaluated in EWAS but may also need further development from statisticians to determine which heterogeneity metrics are most useful.

Most of the EWAS presented here used typical ‘frequentist’ or parametric approaches to data analysis. However, nonparametric approaches such as the use of Bayesian models are also possible and may provide alternative ways to analyze data with less dependency on sample size. Other less traditional statistical approaches should also be considered for these complex datasets, particularly when multiple exposures are addressed. Furthermore, consideration should be given to the evaluation of differentially methylated regions and gene sets rather than single CpG sites in order to reduce the dimensionality of the data and multiple testing burden, as well as to identify more biologically relevant units of methylation change.

The Illumina Infinium HumanMethylationEPIC array is now superseding the HumanMethylation450 Beadchip, targeting 850,000 CpG sites across the genome [83]. This array covers >90% of CpGs on the 450K array, with the addition of CpGs located in enhancer regions. It will be of interest to investigate maternal smoking and micronutrients in relation to methylation on this array, given the upsurge in findings after progressing from the 27K and 450K. Furthermore, we must realize the value of studies investigating the use of whole-genome bisulfite sequencing in this context [10], and RNA sequence data for integrating methylation with gene expression.

Most large-scale epigenetic studies in humans measure methylation in blood. Cross-tissue analyses should also continue as this field develops, and the extent to which methylation signals persist into later childhood and adulthood, which may provide additional insight into widespread and long-term effects of early life environmental exposures on the methylation. Efforts in the context of a consortium-based approaches such as PACE will be valuable for interpreting widespread exposure effects and set a model for evaluating long-term epigenetic effects of other early life environmental exposures.

The downstream health implications of persistent methylation change in response to intra-uterine smoke exposure also require further evaluation. As previous studies have alluded to, persistent changes in DNA methylation might mediate at least some of the associations between smoke exposure in pregnancy and later life health outcomes. It is possible that alterations in genes uncovered by this research may be involved in the mechanisms by which these exposures have lasting effects on children’s health, and by better understanding these mechanisms we can develop more ways of intervening to correct any long-term detrimental effects.

Research in animal models should also continue and be compared with the results of human studies. Collaborations between animal and human researchers will also be helpful in addition to the larger consortium efforts for human studies, in order to test putative causal candidates and proposed mechanisms in different contexts.

What was not presented in this review was the potential combined effect of maternal smoking and micronutrient exposure in pregnancy. Although we did not specifically search for this in our literature review, no studies we identified evaluated a combined effect of maternal smoking and micronutrient exposure. It is possible that these exposures could have potentially opposing impact on the epigenome given the variable effects on offspring phenotype. However, we did not observe overlap in the implicated genes and pathways from these studies so it may also be plausible that they act independently on the developing epigenome. The use of analytic tools such as meta-analysis may shed more light on how the combined effect of these exposures impact epigenome-wide methylation and health end points over time in exposed offspring. Such tools may also be used to detect heterogeneity and bias in contributing studies. Further research (particularly longitudinal periconceptional/perinatal studies) may be able to more clearly elucidate how this combination, and other relevant combinations of exposures or ‘mixtures’ of environmental exposures, cumulatively impact the developing epigenome.

**Executive summary**

**Maternal smoking & the offspring epigenome**

Maternal smoke exposure has a profound effect on epigenome-wide methylation changes in the offspring.
Alterations in DNA methylation are one possible mechanism mediating the harmful effects of smoke exposure.
Epigenome-wide approaches for investigating prenatal smoke exposure have identified an increasing number of methylation signatures in biologically relevant regions of the genome.
An amassing number of epigenome-wide association studies (EWAS) for maternal smoking has led to the identification of a plethora of strong, highly replicated methylation signatures.

**Maternal micronutrient intake & the offspring epigenome**

Micronutrients particularly relevant to epigenetic mechanisms include those involved in the one-carbon metabolism pathway, including folate, choline, betaine and other B vitamins, which provide methyl groups for a range of biochemical reactions including methylation of DNA.
Differences in genome-wide DNA methylation in newborns have been evaluated in relation to maternal folate and other micronutrient exposures in candidate gene methylation studies, although their evaluation in genome-wide association DNA methylation studies is lagging behind.
A comparison of effects in nourished compared with undernourished populations remains to be fully elucidated, as well as the extent to which single micronutrients or combinations of micronutrients impact methylation differences.

**Comparison of EWAS findings**

Stronger statistical significance (in terms of the number of EWAS-significant sites) was observed for the maternal smoking studies compared with the micronutrient studies, and methylation sites were more consistently replicated between studies.

**Statistical methodologies**

Low statistical power, publication bias, noise in the exposure variable, as well as various confounding factors threaten the detection of biological signals of interest and the ability to infer causality from associations identified in relation to maternal smoke and micronutrient exposure.
Methods for improving the robustness of EWAS findings have been implemented in some of these reviewed studies, predominantly in relation to maternal smoking, although we highlight areas for improvement: investigating the magnitude and persistence of effects on methylation, improving power for detecting methylation signals, careful exposure assessment, deeper understanding of cell type heterogeneity, assessing causality with respect to both the maternal exposure and in relation to the observed methylation changes in mediating later health outcomes.
We found the literature describing the influence of maternal smoking in pregnancy on the offspring DNA methylation to be substantially more advanced with regard to use of the above methods compared with the micronutrient literature.

**Other prenatal influences & the offspring epigenome**

The impact of other intrauterine exposures on the epigenome was outside the scope of this review but include: other micronutrients (fatty acids, vitamins and minerals), maternal stress, endocrine disruptors and heavy metals, which have been reviewed in detail elsewhere in this *Epigenomics* issue.
In addition, this review did not specifically address the impact of paternal exposures on the offspring epigenome, which have been implicated in relation to early life programming, and for which some methylation changes have been observed.

**Future perspective**

Additional assessment of micronutrients should be evaluated in larger sample sizes and combined efforts to more adequately compare with folate findings.
Collaborative research efforts such as meta-analysis across multiple cohorts are important to address hypotheses with adequate statistical power.
Thorough consideration should be given to confounding in EWAS studies by factors such as socio-economic position, dietary intake, stress and ethnicity.
Further statistical and causal inference analyses are required to fully elucidate the epigenetic effects of both maternal smoking and micronutrients, given the recent technological developments in methylation arrays.
Cross-tissue analyses are needed to evaluate heterogeneity of effects across sample type and cell type-specific effects should be investigated to extract biological relevance of methylation changes.
More research into the widespread (cross-tissue) and long-term effect of these exposures on offspring methylation is warranted.
Further investigation of the downstream health implications of methylation change in response to intra-uterine exposure also requires further evaluation.
Ongoing efforts in animal studies should continue with the human studies for comparison and validation purposes.
Combined exposures or ‘mixtures’ of exposures should be considered in the context of well-powered and well-described mature datasets.

## References

[1] Bibikova M, Barnes B, Tsan C (2011). High density DNA methylation array with single CpG site resolution. *Genomics*.

[2] Moritsugu KP (2007). The 2006 Report of the Surgeon General: the health consequences of involuntary exposure to tobacco smoke. *Am. J. Prev. Med.*.

[3] Lee KW, Pausova Z (2013). Cigarette smoking and DNA methylation. *Front. Genet.*.

[4] Suter MA, Anders AM, Aagaard KM (2013). Maternal smoking as a model for environmental epigenetic changes affecting birthweight and fetal programming. *Mol. Hum. Reprod.*.

[5] Joubert BR, Felix JF, Yousefi P (2016). DNA methylation in newborns and maternal smoking in pregnancy: genome-wide consortium meta-analysis. *Am. J. Hum. Genet.*.

[6] Black RE, Allen LH, Bhutta ZA (2008). Maternal and child undernutrition: global and regional exposures and health consequences. *Lancet*.

[7] Anderson OS, Sant KE, Dolinoy DC (2012). Nutrition and epigenetics: an interplay of dietary methyl donors, one-carbon metabolism and DNA methylation. *J. Nutr. Biochem.*.

[8] Van Mil NH, Bouwland-Both MI, Stolk L (2014). Determinants of maternal pregnancy one-carbon metabolism and newborn human DNA methylation profiles. *Reproduction*.

[9] Alexander M, Karmaus W, Holloway JW (2013). Effect of GSTM2–5 polymorphisms in relation to tobacco smoke exposures on lung function growth: a birth cohort study. *BMC Pulm. Med.*.

[10] Bauer T, Trump S, Ishaque N (2016). Environment-induced epigenetic reprogramming in genomic regulatory elements in smoking mothers and their children. *Mol. Syst. Biol.*.

[11] Breton CV, Siegmund KD, Joubert BR (2014). Prenatal tobacco smoke exposure is associated with childhood DNA CpG methylation. *PLoS ONE*.

[12] Chhabra D, Sharma S, Kho AT (2014). Fetal lung and placental methylation is associated with in utero nicotine exposure. *Epigenetics*.

[13] De Vocht F, Simpkin AJ, Richmond RC, Relton C, Tilling K (2015). Assessment of offspring DNA methylation across the lifecourse associated with prenatal maternal smoking using Bayesian Mixture Modelling. *Int. J. Environ. Res. Public Health*.

[14] Ivorra C, Fraga MF, Bayon GF (2015). DNA methylation patterns in newborns exposed to tobacco *in utero*. *J. Transl. Med.*.

[15] Joubert BR, Haberg SE, Nilsen RM (2012). 450K epigenome-wide scan identifies differential DNA methylation in newborns related to maternal smoking during pregnancy. *Environ. Health Perspect.*.

[16] Joubert BR, Haberg SE, Bell DA (2014). Maternal smoking and DNA methylation in newborns: in utero effect or epigenetic inheritance?. *Cancer Epidemiol. Biomarkers Prev.*.

[17] Kupers LK, Xu X, Jankipersadsing SA (2015). DNA methylation mediates the effect of maternal smoking during pregnancy on birthweight of the offspring. *Int. J. Epidemiol.*.

[18] Ladd-Acosta C, Shu C, Lee BK (2016). Presence of an epigenetic signature of prenatal cigarette smoke exposure in childhood. *Environ. Res.*.

[19] Lee KW, Richmond R, Hu P (2015). Prenatal exposure to maternal cigarette smoking and DNA methylation: epigenome-wide association in a discovery sample of adolescents and replication in an independent cohort at birth through 17 years of age. *Environ. Health. Perspect.*.

[20] Maccani JZ, Koestler DC, Houseman EA, Marsit CJ, Kelsey KT (2013). Placental DNA methylation alterations associated with maternal tobacco smoking at the RUNX3 gene are also associated with gestational age. *Epigenomics*.

[21] Markunas CA, Xu Z, Harlid S (2014). Identification of DNA methylation changes in newborns related to maternal smoking during pregnancy. *Environ. Health Perspect.*.

[22] Ray MA, Tong X, Lockett GA, Zhang HM, Karmaus WJJ (2016). An efficient approach to screening epigenome-wide data. *Biomed. Res. Int.*.

[23] Reese SE, Zhao S, Wu MC (2016). DNA methylation score as a biomarker in newborns for sustained maternal smoking during pregnancy. *Environ. Health Perspect.*.

[24] Richmond RC, Simpkin AJ, Woodward G (2015). Prenatal exposure to maternal smoking and offspring DNA methylation across the lifecourse: findings from the Avon Longitudinal Study of Parents and Children (ALSPAC). *Hum. Mol. Genet.*.

[25] Rzehak P, Saffery R, Reischl E (2016). Maternal smoking during pregnancy and DNA-methylation in children at age 5.5 years: epigenome-wide-analysis in the European Childhood Obesity Project (CHOP)-study. *PLoS ONE*.

[26] Sanders AP, Smeester L, Rojas D (2014). Cadmium exposure and the epigenome: exposure-associated patterns of DNA methylation in leukocytes from mother-baby pairs. *Epigenetics*.

[27] Suderman M, Pappas JJ, Borghol N (2015). Lymphoblastoid cell lines reveal associations of adult DNA methylation with childhood and current adversity that are distinct from whole blood associations. *Int. J. Epidemiol.*.

[28] Suter M, Ma J, Harris A (2011). Maternal tobacco use modestly alters correlated epigenome-wide placental DNA methylation and gene expression. *Epigenetics*.

[29] Wang IJ, Chen SL, Lu TP, Chuang EY, Chen PC (2013). Prenatal smoke exposure, DNA methylation, and childhood atopic dermatitis. *Clin. Exp. Allergy*.

[30] Xu Z, Niu L, Li L, Taylor JA (2016). ENmix: a novel background correction method for Illumina HumanMethylation450 BeadChip. *Nucleic Acids Res.*.

[31] Yang SI, Kim BJ, Lee SY (2015). Prenatal Particulate Matter/Tobacco Smoke Increases Infants’ Respiratory Infections: COCOA Study. *Allergy Asthma Immunol. Res.*.

[32] Ray MA, Tong X, Lockett GA, Zhang H, Karmaus WJ (2016). An efficient approach to screening epigenome-wide data. *Biomed. Res. Int.*.

[33] Houseman EA, Accomando WP, Koestler DC (2012). DNA methylation arrays as surrogate measures of cell mixture distribution. *BMC Bioinformatics*.

[34] Denison MS, Nagy SR (2003). Activation of the aryl hydrocarbon receptor by structurally diverse exogenous and endogenous chemicals. *Annu. Rev. Pharmacol. Toxicol.*.

[35] Yoon D, Kim YJ, Cui WY (2012). Large-scale genome-wide association study of Asian population reveals genetic factors in FRMD4A and other loci influencing smoking initiation and nicotine dependence. *Hum. Genet.*.

[36] Zeilinger S, Kuhnel B, Klopp N (2013). Tobacco smoking leads to extensive genome-wide changes in DNA methylation. *PLoS ONE*.

[37] Joehanes R, Just AC, Marioni RE (2016). Epigenetic signatures of cigarette smoking. *Circ. Cardiovasc. Genet.*.

[38] Monick MM, Beach SR, Plume J (2012). Coordinated changes in AHRR methylation in lymphoblasts and pulmonary macrophages from smokers. *Am. J. Med. Genet. B Neuropsychiatr. Genet.*.

[39] Suter M, Abramovici A, Showalter L (2010). In utero tobacco exposure epigenetically modifies placental CYP1A1 expression. *Metabolism*.

[40] Shenker NS, Polidoro S, Van Veldhoven K (2013). Epigenome-wide association study in the European Prospective Investigation into Cancer and Nutrition (EPIC-Turin) identifies novel genetic loci associated with smoking. *Hum. Mol. Genet.*.

[41] Zudaire E, Cuesta N, Murty V (2008). The aryl hydrocarbon receptor repressor is a putative tumor suppressor gene in multiple human cancers. *J. Clin. Invest.*.

[42] Teschendorff AE, Yang Z, Wong A (2015). Correlation of smoking-associated DNA methylation changes in buccal cells with DNA methylation changes in epithelial cancer. *JAMA Oncol.*.

[43] Cnattingius S (2004). The epidemiology of smoking during pregnancy: smoking prevalence, maternal characteristics, and pregnancy outcomes. *Nicotine Tob. Res.*.

[44] Amarasekera M, Martino D, Ashley S (2014). Genome-wide DNA methylation profiling identifies a folate-sensitive region of differential methylation upstream of ZFP57-imprinting regulator in humans. *FASEB J.*.

[45] Binder AM, Michels KB (2013). The causal effect of red blood cell folate on genome-wide methylation in cord blood: a Mendelian randomization approach. *BMC Bioinformatics*.

[46] Emes RD, Clifford H, Haworth KE (2013). Antiepileptic drugs and the fetal epigenome. *Epilepsia*.

[47] Gonseth S, De Smith AJ, Roy R (2016). Genetic contribution to variation in DNA methylation at maternal smoking sensitive loci in exposed neonates. *Epigenetics*.

[48] Joubert BR, Den Dekker HT, Felix JF (2016). Maternal plasma folate impacts differential DNA methylation in an epigenome-wide meta-analysis of newborns. *Nat. Commun.*.

[49] Khulan B, Cooper WN, Skinner BM (2012). Periconceptional maternal micronutrient supplementation is associated with widespread gender related changes in the epigenome: a study of a unique resource in the Gambia. *Hum. Mol. Genet.*.

[50] Mozhui K, Smith AK, Tylavsky FA (2015). Ancestry dependent DNA methylation and influence of maternal nutrition. *PLoS ONE*.

[51] Rakyan VK, Down TA, Balding DJ, Beck S (2011). Epigenome-wide association studies for common human diseases. *Nat. Rev. Genet.*.

[52] Paul DS, Beck S (2014). Advances in epigenome-wide association studies for common diseases. *Trends Mol. Med.*.

[53] Lin X, Barton S, Holbrook JD (2016). How to make DNA methylome wide association studies more powerful. *Epigenomics*.

[54] Birney E, Davey Smith G, Greally JM (2016). Epigenome-wide association studies and the interpretation of disease -omics. *PLoS Genet.*.

[55] Cecil CA, Walton E, Smith RG (2016). DNA methylation and substance-use risk: a prospective, genome-wide study spanning gestation to adolescence. *Transl. Psychiatry*.

[56] Reik W (2007). Stability and flexibility of epigenetic gene regulation in mammalian development. *Nature*.

[57] Czeizel AE, Dudas I (1992). Prevention of the first occurrence of neural-tube defects by periconceptional vitamin supplementation. *N. Engl. J. Med.*.

[58] Waterland RA, Michels KB (2007). Epigenetic epidemiology of the developmental origins hypothesis. *Annu. Rev. Nutr.*.

[59] Relton CL, Davey Smith G (2010). Epigenetic epidemiology of common complex disease: prospects for prediction, prevention, and treatment. *PLoS Med.*.

[60] Jaffe AE, Irizarry RA (2014). Accounting for cellular heterogeneity is critical in epigenome-wide association studies. *Genome Biol.*.

[61] Bauer M, Linsel G, Fink B (2015). A varying T cell subtype explains apparent tobacco smoking induced single CpG hypomethylation in whole blood. *Clin. Epigenetics*.

[62] Bauer M, Fink B, Thurmann L, Eszlinger M, Herberth G, Lehmann I (2015). Tobacco smoking differently influences cell types of the innate and adaptive immune system-indications from CpG site methylation. *Clin. Epigenetics*.

[63] Bakulski KM, Feinberg JI, Andrews SV (2016). DNA methylation of cord blood cell types: applications for mixed cell birth studies. *Epigenetics*.

[64] Houseman EA, Molitor J, Marsit CJ (2014). Reference-free cell mixture adjustments in analysis of DNA methylation data. *Bioinformatics*.

[65] Bell JT, Pai AA, Pickrell JK (2011). DNA methylation patterns associate with genetic and gene expression variation in HapMap cell lines. *Genome Biol.*.

[66] Gaunt TR, Shihab HA, Hemani G (2016). Systematic identification of genetic influences on methylation across the human life course. *Genome Biol.*.

[67] Chen YA, Lemire M, Choufani S (2013). Discovery of cross-reactive probes and polymorphic CpGs in the Illumina Infinium HumanMethylation450 microarray. *Epigenetics*.

[68] Teschendorff AE, Zhuang J, Widschwendter M (2011). Independent surrogate variable analysis to deconvolve confounding factors in large-scale microarray profiling studies. *Bioinformatics*.

[69] Heijmans BT, Tobi EW, Stein AD (2008). Persistent epigenetic differences associated with prenatal exposure to famine in humans. *Proc. Natl Acad. Sci. USA*.

[70] Waterland RA, Kellermayer R, Laritsky E (2010). Season of conception in rural Gambia affects DNA methylation at putative human metastable epialleles. *PLoS Genet.*.

[71] Richmond RC, Al-Amin A, Davey Smith G, Relton CL (2014). Approaches for drawing causal inferences from epidemiological birth cohorts: a review. *Early Hum. Dev.*.

[72] Lipsitch M, Tchetgen Tchetgen E, Cohen T (2010). Negative controls: a tool for detecting confounding and bias in observational studies. *Epidemiology*.

[73] Davey Smith G (2012). Negative control exposures in epidemiologic studies. *Epidemiology*.

[74] Davey Smith G, Ebrahim S (2003). ‘Mendelian randomization’: can genetic epidemiology contribute to understanding environmental determinants of disease?. *Int. J. Epidemiol.*.

[75] Davey Smith G, Hemani G (2014). Mendelian randomization: genetic anchors for causal inference in epidemiological studies. *Hum. Mol. Genet.*.

[76] Davey Smith G, Lawlor DA, Harbord R, Timpson N, Day I, Ebrahim S (2007). Clustered environments and randomized genes: a fundamental distinction between conventional and genetic epidemiology. *PLoS Med.*.

[77] Davey Smith G (2008). Assessing intrauterine influences on offspring health outcomes: can epidemiological studies yield robust findings?. *Basic Clin. Pharmacol. Toxicol.*.

[78] Price EM, Penaherrera MS, Portales-Casamar E (2016). Profiling placental and fetal DNA methylation in human neural tube defects. *Epigenet. Chromatin*.

[79] Talens RP, Boomsma DI, Tobi EW (2010). Variation, patterns, and temporal stability of DNA methylation: considerations for epigenetic epidemiology. *FASEB J.*.

[80] Richmond RC, Hemani G, Tilling K, Davey Smith G, Relton CL (2016). Challenges and novel approaches for investigating molecular mediation. *Hum. Mol. Genet.*.

[81] Relton CL, Davey Smith G (2012). Two-step epigenetic Mendelian randomization: a strategy for establishing the causal role of epigenetic processes in pathways to disease. *Int. J. Epidemiol.*.

[82] Morales E, Vilahur N, Salas LA (2016). Genome-wide DNA methylation study in human placenta identifies novel loci associated with maternal smoking during pregnancy. *Int. J. Epidemiol.*.

[83] Moran S, Arribas C, Esteller M (2016). Validation of a DNA methylation microarray for 850,000 CpG sites of the human genome enriched in enhancer sequences. *Epigenomics*.

