# Supplementary material for: Contrasting the effects of intra-uterine smoking and one-carbon micronutrient exposures on offspring DNA methylation
Source: Epigenomics. 2017 Feb 17;9(3):351–67. doi: 10.2217/epi-2016-0135 (PMC5331918; doi:10.2217/epi-2016-0135)
Supplement: Supplementary file 1 [file epi-09-351-s1.docx]

**Supplemental Methods**

We searched the literature (PubMed, Scopus, and Web of Science databases) in September 2016, to identify novel publications describing maternal smoking or micronutrient exposures during pregnancy and the offspring epigenome. The micronutrients included in our search were those identified to play important roles in one-carbon metabolism [7]: Folate, choline, betaine, methionine, vitamin B2 (riboflavin), B6, B12 (cobalamin), and homocysteine. The search strategy is detailed below.

**Original Searches**

***Smoking - PubMed Search: 208 results***

(((((((((((("in utero"[All Fields] OR prenatal[All Fields] OR antenatal[All Fields] OR perinatal[All Fields] OR preconception[All Fields] OR pregnant[All Fields] OR mother[All Fields] OR fetus[All Fields] OR fetal[All Fields])) OR ("Maternal Exposure"[Mesh] OR "Prenatal Exposure Delayed Effects"[Mesh] OR "Pregnancy"[Mesh]))) AND ((("Epigenomics"[Mesh] OR "Epigenesis, Genetic"[Mesh] OR "DNA Methylation"[Mesh])) OR (epigenetic[Title/Abstract] OR epigenome[Title/Abstract] OR epigenomics[Title/Abstract]))) AND (((smoking[All Fields] OR "tobacco"[All Fields] OR cigarette[All Fields])) OR ("Smoke"[Mesh] OR "Tobacco Smoke Pollution"[Mesh] OR "Tobacco/adverse effects"[Mesh])))))))))

***Smoking - Web of Science Search: 247***

TS=("in utero" OR prenatal OR antenatal OR perinatal OR preconception OR fetus OR fetal OR pregnan*) AND TS=((maternal OR tobacco OR cigarette) NEAR/2 smok*) AND TS=(epigenom* OR epigenetic* OR "DNA methylation") NOT TS=miRNA

***Smoking - Scopus Search: 263***

TITLE-ABS-KEY((maternal OR tobacco OR cigarette) PRE/2 smok*) AND TITLE-ABS-KEY( "in utero" OR prenatal OR antenatal OR perinatal OR preconception OR fetus OR fetal OR pregnan*) AND TITLE-ABS-KEY(epigenom* OR epigenetic* OR "DNA methylation") AND NOT TITLE-ABS-KEY(miRNA) AND ( LIMIT-TO(LANGUAGE,"English" ) ) AND ( EXCLUDE(DOCTYPE,"le" ) OR EXCLUDE(DOCTYPE,"er" ) )

***Micronutrients - PubMed: 167***

((((((((((((((((pregnancy OR maternal OR pregnant OR gestation OR mother))) AND ((((epigenetic[Title/Abstract] OR epigenome[Title/Abstract] OR epigenomics[Title/Abstract] OR "dna methylation"[Title/Abstract]))) OR (("Epigenomics"[Mesh] OR "Epigenesis, Genetic"[Mesh] OR "DNA Methylation"[Mesh])))) AND (("Folate" OR "Folic Acid" OR Choline OR Betaine OR Methionine OR B2 OR Riboflavin OR B6 OR "Vitamin B 6"[Mesh] OR B12 OR "Vitamin B 12"[Mesh] OR Homocysteine OR "micronutrient" OR "micronutrients"))))))) AND ("in utero" OR prenatal OR perinatal OR antenatal OR fetus OR fetal OR exposure OR "Maternal Nutritional Physiological Phenomena"[Mesh])))) AND Humans[Mesh] AND English[lang]))))

***Micronutrients - Web of Science: 356***

TS=(pregnan* OR maternal OR mother* OR gestation*) AND TS=(epigen* OR "DNA Methylation") AND TS=(Folate OR "folic acid” OR Choline OR Betaine OR Methionine OR B2 OR Riboflavin OR B6 OR B12 OR Homocysteine OR micronutrient*) AND TS=(fetal OR "in utero" OR fetus OR *natal) NOT TI=(mouse OR mice OR "rat" OR rats OR piglet OR swine OR "pig" OR "pigs" OR chick OR "cow" OR cows OR horse* OR baboon* OR monkey* OR sheep OR "dams")

Refined by: LANGUAGES: ( ENGLISH ) AND [excluding] DOCUMENT TYPES: ( REFERENCE MATERIAL OR NEWS OR DATA SET )

***Micronutrients - Scopus: 335***

TITLE-ABS-KEY ( pregnan*  OR  maternal  OR  mother*  OR  gestation* )  AND  TITLE-ABS-KEY ( epigen*  OR  "DNA Methylation" )  AND  TITLE-ABS-KEY ( folate  OR  "folic acid"  OR  choline  OR  betaine  OR  methionine  OR  b2  OR  riboflavin  OR  b6  OR  b12  OR  homocysteine  OR  micronutrient* )  AND  TITLE-ABS-KEY ( fetal  OR  "in utero"  OR  fetus  OR  *natal )  AND NOT  TITLE ( mouse  OR  mice  OR  "rat"  OR  rats  OR  piglet  OR  swine  OR  "pig"  OR  "pigs"  OR  chick  OR  "cow"  OR  cows  OR  horse*  OR  baboon*  OR  monkey*  OR  sheep  OR  "dams" )  AND  ( LIMIT-TO ( LANGUAGE ,  "English" ) )  AND  ( LIMIT-TO ( DOCTYPE ,  "ar" )  OR  LIMIT-TO ( DOCTYPE ,  "re" )  OR  LIMIT-TO ( DOCTYPE ,  "cp" )  OR  LIMIT-TO ( DOCTYPE ,  "ed" )  OR  LIMIT-TO ( DOCTYPE ,  "ch" )  OR  LIMIT-TO ( DOCTYPE ,  "ip" ) )

**Filtering and an updated search was completed on 8/15/16:**

Filtering: We removed papers with the following key terms: Alu, repeat, LINE-1, global DNA methylation or global methylation, animal, mouse, mice, rodent, zebrafish (anything not human) removed. Removed books, book chapters, editorials, meeting abstracts, and related. Key terms were included to search for genome-wide or epigenome-wide DNA Methylation studies: 450K, Methyl450, Methylation450, Beadchip, Illumina, Infinium 450K, EPIC, 800K.

If re-searched the following terms should be added to the above: AND (450K OR Methyl450 OR Methylation450 OR beadchip OR "bead chip" OR 800k OR epic OR EWAS OR genome-wide OR genomewide OR epigenome-wide OR epigenomewide)

Supplementary Table 1. Studies identified in literature review evaluating maternal smoking exposure and offspring epigenome-wide DNA methylation

| **Study** | **PubMed ID** | **Subjects (n)** | **Measure of exposure** | **Technology^1^** | **Tissue for methylation** | **Timepoint of offspring outcome** | **Methods** | **Key findings** |
| --- | --- | --- | --- | --- | --- | --- | --- | --- |
| Alexander et al, 2013 | 24004509 | 245 18 year olds | Questionnaire report of maternal smoking during pregnancy at enrolment and postpartum and urinary cotinine | 450K | Whole blood samples collected at age 18 | 18 years | EWAS using linear mixed modelling and GSTM2×in utero smoke exposure interaction model. Adjusted for current smoking, second hand smoke exposure, BMI, height, *GSTM1.* | No statistically significant findings for maternal smoking alone during pregnancy on offspring DNA methylation alone. Suggestive replication of maternal smoking X *GSTM2* mutation interaction on offspring methylome. |
| Bauer et al, 2016 | 27013061 | 16 mother-child pairs for main analysis. Targeted methylation analysis in entire cohort (n=471) for validation. | Determined based on self-reported status and urine cotinine levels >100 ug/g creatinine | Whole-genome bisulphite sequencing | Blood samples | Birth, age 1 and age 4 | Mapped DNA methylation, histone modifications and gene expression. Separated environment-associated from genotype-related changes. Longitudinal analysis from birth until 4 years. Adjusted for cellular composition. | 8,409 significant DMRs in offspring at birth, 1,404 non-genetic. Enrichment of differential methylation in enhancer elements. Hyperactive chromatin states in children exposed. Evidence for persistence of methylation signatures over years of life. 82% of all non-genetic DMRs were in same methylation state 1 year after birth. Some evidence for mediation of *JNK2* methylation linking maternal smoking with impaired lung function in childhood. |
| Breton et al, 2014 | 24964093 | 527 mother-offspring pairs | Retrospectively reported maternal smoking in pregnancy | 27K | Whole blood | 5-12 years | EWAS (using beta regression) of maternal smoking within asthmatic children. Adjusted for age, gender and clinical site. Further adjustment for childhood smoke exposure. Replication in two additional cohorts and mRNA expression analysis. | 19 CpG sites associated with prenatal smoke at FDR<0.05. *FRDM4A* and *Cllorf52* replicated in two independent populations, irrespective of asthmatic status and timing of sample collection. No significant associations between methylation and expression for tested genes. |
| Chhabra et al, 2014 | 25482056 | 85 fetal lung tissue and 80 placental tissue samples | Placental cotinine as a marker of nicotine exposure | 450K | Lung and placental tissue | Post-conception | EWAS of placental cotinine and methylation in lung and placental tissue, investigated adjustment for surrogate variables and post-conceptional age. Assessed concordance between tissues. Pyrosequencing of selected sites for validation. Gene set enrichment analysis. | 264 CpGs in lung tissue associated with cotinine with P<0.05 but 0 after adjustment for multiple testing. 657 CpGs in placental tissue associated with cotinine with P<0.05 and 2 with a Benjamini-Hochberg P<0.05. Sites associated in fetal lung analysis mapped to *PKP3*, *ANKRD33B, CNTDN2* and *DPP10*. In placenta, sites mapped to *GTF2H2C* and *GTF2H2D*. 101 CpG sites concordant between lung and placental tissue (p<0.05). Enrichment of specific disorders e.g. asthma and immune disorders. |
| De Vocht et al, 2015 | 26580635 | 790 mother-child pairs | Self-reported smoking status of mother in pregnancy | 450K | Cord blood and peripheral blood | Birth, 7 and 17 years | Re-analysis of data from PMID 25552657 using hierarchical Bayesian Mixture Modelling to address correlation between CpG sites and to investigate longitudinal methylation. Adjusted for maternal age, pre-pregnancy BMI, pre-pregnancy weight, parity, educational attainment, social class, alcohol intake, paternal smoking and top 20 surrogate variables. | The use of Bayesian Mixture Modelling could be a useful addition to the statistical methodologies to analyse epigenetic data sets without the need for pre-analysis data reduction steps in downstream analyses. |
| Ivorra et al, 2015 | 25623364 | 20 mother-offspring pairs | Self-reported maternal smoking throughout pregnancy, confirmed by umbilical cord blood cotinine levels | Global DNA methylation assay using ELISA and 450K | Cord blood | Birth | EWAS of maternal smoking (n=10 exposed) and offspring cord methylation in the absence of fetal growth restriction. Adjusted for offspring sex, maternal age and cellular composition. | Global hypomethylation among exposed but 31 CpG sites in 25 gene regions, for which 90.3% exhibited higher methylation levels in exposed group and largely in CpG islands. Top site located in *ADM* gene. |
| Joubert et al, 2012 | 22851337 | 1,062 mother-offspring pairs; replication in 36 mother-offspring pairs | Plasma cotinine measured at 18 weeks of gestation; self-reported smoking | 450K | Cord blood | Birth | EWAS of maternal plasma cotinine. Assessment of dose response and associations with self-reported smoking. Adjusted for maternal age, maternal education, parity and asthma status. | Differential methylation at 26 CpG sites mapping to 10 genes at Bonferroni significance. 8 CpGs in *GFI1*, 4 CpGs in *AHRR*, 4 CpGs in *CYP1A1*, 4 CpGs in *MYO1G*. Dose response for cotinine and methylation. Fewer Bonferroni-significant signals with self-reported smoking. Replicated findings for CpGs in *AHRR*, *CYP1A1* and *GFI1* at Bonferroni-correction in an independent data set. |
| Joubert et al, 2014 | 24740201 | 1,042 mother-offspring pairs | Plasma cotinine measured at 18 weeks of gestation; self-reported smoking in pregnancy | 450K | Cord blood | Birth | Evaluated the impact of timing of maternal smoking, paternal smoking and grandmaternal smoking at 26 CpG loci previously associated with prenatal smoking in cord blood (PMID 22851337). Adjusted for maternal age, maternal education and parity. | Found that signals reflect sustained (gestational week 18), rather than short-term, exposure. No evidence for differential methylation due to smoking by mother prior to pregnancy or before week 18, father’s smoking before conception or grandmother’s smoking i.e. overall supporting in utero exposure rather than epigenetic inheritance |
| Joubert et al, 2016 | 27040690 | 6,685 across 13 cohorts | Determined based on self-reported smoking in the 13 cohorts with harmonized variables for “sustained” and “any” smoking during pregnancy | 450K | Cord blood and peripheral blood | Birth, childhood in some studies (5 cohorts, n=3,187) | Evaluated both cord blood methylation and peripheral blood in childhood. Separate models to evaluate both sustained smoking and any smoking during pregnancy using robust linear regression. Adjusted for maternal age, maternal education, parity.Adjusted for second-hand tobacco smoke in older children. Further model adjusted for estimated cell type proportion and excluding non-European cohorts. Investigated associations with gene expression. | 568 Bonferroni-significant and 6073 FDR (0.05) CpG sites associated with sustained smoking, including 2965 CpGs in 2,017 gene regions not previously related to smoking and methylation. Results robust to different normalization methods and cell type adjustment. Observed enrichment in pathways and processes critical to development and conditions that can be caused by maternal smoking e.g. orofacial clefts, asthma. Methylation at a number of CpGs associated with with gene expression. Many sites with persistence into later childhood (assessed in 5 cohorts, n=3187). |
| Kupers et al, 2015 | 25862628 | 255 in discovery, 678 and 745 in replication cohorts | Self-reported maternal and paternal smoking during pregnancy | 450K | Cord blood | Birth | EWAS of maternal smoking and methylation, adjusted for offspring sex, gestational age, maternal age, pre-pregnancy BMI, educational level, cell type composition. Mediation analysis for effect of smoking-induced methylation changes on birthweight using Baron and Kenny approach and causal inference methods. Replication and meta-analysis of mediation results from two independent birth cohorts. Functional network and enrichment analysis. | 35 CpG sites differentially methylated at FDR < 0.05, of which 23 survived Bonferroni correction. CpG sites at *GFI1* shown to partially mediate the effect of maternal smoking on birthweight, explaining 12-19% effect of smoking on lower birthweight. Functional enrichment analysis suggested activation of cell-mediated immunity. |
| Ladd-Acosta et al, 2016 | 26610292 | 572 children | Retrospective self-report of prenatal smoking in each trimester and overall pregnancy period. Main analysis of exposure in second trimester | 450K | Whole blood | 3-5 years | Assessed methylation at 26 CpG loci previously associated with prenatal smoking in cord blood [PMID 22851337]. Adjusted for sex, race, maternal education, age, cell composition. Also assessed potential confounding by alcohol, medication use and passive smoke exposure. Classification models for DNA methylation as a biomarker for exposure. | Concordance between prenatal smoking at birth and in preschool-aged children but systematically weaker (7/26 sites after multiple testing correction and consistency in direction of effect). Sites in *MYO1G, CYP1A1* and one site at *AHRR* showing increase methylation with exposure whereas *CNTNAP2* and *GFI1* showing decrease. DNA methylation pattern at 26 loci classified prenatal exposure with 81% accuracy. |
| Lee et al, 2015 | 25325234 | 132 in discovery and 447 in replication cohort | Self-reported smoking status determined during second trimester of pregnancy. | 450K | Cord and peripheral blood | 15 years in discovery; birth, 7 and 17 years in replication | EWAS of prenatal smoke exposure during adolescence in discovery cohort (n=132) and at birth, during childhood and during adolescence in replication cohort (n=447). In discovery, 66 exposed adolescents matched to 66 non-exposed. Adjusted for age, sex, cell composition, current and secondhand smoking. | Persistence of methylation alterations into adolescence. 5 CpGs in *MYO1G* and *CNTNAP2* differentially methylated between exposed and nonexposed individuals during adolescence. In both cohorts and all time points, differential methylation in same direction and of a similar magnitude and not altered by adjustment for current smoking. |
| Maccani et al, 2013 | 24283877 | 206 mother-offspring pairs | Smoking status at any time during pregnancy, in recorded from patient charts | 27K | Placenta | Birth | EWAS of maternal smoking in study over-sampled for small-for-gestational age infants. Adjusted for maternal age, gender, birth weight and delivery method. Pyrosequencing of 22 samples for genes with multiple CpG loci residing within them. Investigated RUNX3 CpG sites with gestational age in multivariable model. | 1918 CpG sites associated with maternal smoking at p<0.05 but 0 after correction for multiple testing. 7 loci residing within intronic and promoter regions of *RUNX3* with differential methylation. 1 CpG in *RUNX3* associated with gestational age. |
| Markunas et al, 2014 | 24906187 | 889 mother-offspring pairs | Self-reported smoking during first trimester of pregnancy | 450K | Cord blood | Birth | EWAS of maternal smoking and offspring methylation. Investigated dose response effect of cigarettes smoked per day. Replication in additional data set at a) site level b) gene level. Also considered overlap between smoking-associated methylation in adults and newborns. Adjusted for facial cleft status, infant’s sex, cellular composition, and further sensitivity analysis adjusting for maternal alcohol consumption, education, age, BMI, dietary folate, folic acid supplement use, multivitamin use, parity, gestational age and infant’s birth weight. | 185 CpGs with altered methylation in infants of smokers at genome-wide significance, corresponding to 110 gene regions. 43% CpGs had decreased methylation and 57% had increased. Average percentage change ranging from -8 to 7% with a mean of -2%. 107/185 CpGs showed evidence of a dose-response. Enrichment for CpG shores. 62 CpGs replicated in another study of maternal smoking. 21 CpGs identified with same direction of effect in adult smoking studies (majority with decreased methylation). 10 genes (23 CpGs) with newly established links to maternal smoking e.g. *FRMD4A, ATP9A, CALNT2, MEG3.* |
| Ray et al, 2016 | 27034928 | 245 female offspring | Smoke exposure in pregnancy (yes/no) | 450K | Whole blood | 18 years | SVA analysis to account for latent confounding variables derived in training set and applied to testing set in ttScreening package. Applied to EWAS of maternal smoking. | FDR = 10 sites, Bonferroni = 5 sites, TT screening = 91 sites including 18 previously identified |
| Reese et al, 2015 | 27323799 | 1,057 in training set and 221 in test set | Self-reported smoking status at 17 and 30 weeks gestation; maternal plasma cotinine measured at 18 weeks | 450K | Cord blood | Birth | Development of smoking biomarker by taking top 200 most significant CpGs from EWAS in training set used in a LASSO model with 100 iterations to choose a set of CpGs for generating a smoking score. ROC analysis to determine sensitivity, specificity and accuracy in test set. Also investigated how score relates to birthweight. Models adjusted for gender, gestational age, maternal education, maternal age, parity and selection. | 28 CpGs retained in all 100 iterations in training set, 5 of the original 10 loci from Joubert et al, 2012 identified. In test set, AUC = 0.90 (0.83, 0.97), sensitivity 58%, accuracy 91% and specificity 97%. Performance in evaluating relationship with reduced birth weight similar to cotinine-based and self-reported smoking status. |
| Richmond et al, 2015 | 25552657 | 790 mother-child pairs | Self-reported smoking status of mother in pregnancy (each trimester, cigarettes/day) | 450K | Cord blood and peripheral blood | Birth, 7 and 17 years | Investigated multivariable associations between prenatal exposure to maternal smoking and offspring DNA methylation at three time points, from birth to age 17. Assessed adjustment for maternal age, pre-pregnancy BMI, pre-pregnancy weight, parity, educational attainment, social class, alcohol intake, paternal smoking, cellular composition. Assessed dose-response relationship between duration and intensity of smoking and offspring methylation. Compared maternal smoking with paternal smoking associations with offspring methylation. Performed longitudinal analysis to investigate reversibility/persistence of methylation patterns over time. | Identified 15 CpG sites in seven gene regions associated with maternal smoking in cord blood at Bonferroni significance. Dose-response in relation to both smoking duration and intensity. Evidence for a stronger maternal than paternal effect. Longitudinal analysis demonstrated that some CpG sites showed reversibility in methylation (*GFI1, KLF13, ATP9A)* whereas others showed a persistently perturbed pattern (*AHRR, MYO1G, CYP1A1* and *CNTNAP2*). |
| Rzehak et al, 2016 | 27171005 | 366 | Smoke exposure beyond 12^th^ week gestation i.e. prolonged exposure | 450K | Whole blood (buffy coats) | 5.5 years | EWAS of maternal smoking, adjusted for sex, age, study centre, maternal education, postnatal smoking, cellular composition, top 30 principal components. | Methylation levels at 5 CpG sites in *MYO1G, CNTNAP2* and *FRMD4A* at FDR significance. Decreased methylation for *CNTNAP2* but increased for *MYO1G* and *FRMD4A.* |
| Sanders et al, 2014 | 24169490 | 17 mother-offspring pairs | Maternal blood levels of cadmium and cotinine | Affymetrix Human Promoter 1.0R array (16,000 promoter-based CpG islands) | Cord blood | Birth | Gene-specific methylation analysis of both cadmium and cotinine exposure. Assessed confounding by maternal age, race, parity, sex and birthweight. Enrichment analysis. Validation of methylation assessment with qPCR based methylation in a gene-specific analysis. | 61 cadmium methylation gene sets, 366 cotinine methylation gene sets, 30 overlapping methylation gene sets. Overall, majority of differentially methylated genes showed hypermethylation with Cd exposure. Enrichment analysis showed most enriched biological function categories were gene expression, cell cycle, cell death and nervous system development. |
| Suderman et al, 2015 | 26351305 | 42 individuals with lymphoblastoid cell line (LCL) profiles, 30 with whole blood (WB) profiles, 28 with both | Self-reported smoking status in pregnancy | 450K | LCLs and WB | 45 years | Differentially methylated sites were identified using modified t-tests. Differentially methylated regions were identified using the global test R package. Matched on age, sex, and socio-economic position. | 1 DMR in WB associated with prenatal tobacco at FDR < 0.2. No single sites or LCL associations at FDR < 0.2 and for methylation difference > 1%. |
| Suter et al, 2011 | 21937876 | 36 mother offspring pairs (18 smokers, 18 non-smokers) | Self-reported smoking status | 27K | Placenta | Birth | Side-by-side gene transcription with methylation arrays to interrogate correlative changes associated with maternal smoke exposure. Matching for maternal age, race, BMI and gestational age. Validation of both expression and methylation analysis. Pathway analysis. Regression analysis of smoking and birth weight interaction. | Expression of 623 genes and methylation of 1024 CpG sites are significantly altered among smokers. Validation of top 5 CpG sites. Correlation of transcription and methylation in 25 genes among non-smokers but 438 genes among smokers, with dominant effect in oxidative stress pathways. Differential methylation at 6 CpGs attributed to smoking status and infant weight interactions. |
| Wang et al, 2013 | 23600544 | 14 mother-offspring pairs. Validation in larger sample of 150 mother-offspring pairs. | Cord blood cotinine | 27K | Cord blood | Birth | Analysed DNA methylation of gestational age and gender-matched offspring of high (n=7) and low exposure (n=7). Validation using methylation-dependent fragment separation in larger sample. Looked at expression of differentially methylated sites. Took forward candidate genes with CpG islands in the promoter regions and reported associations with smoke exposure and/or atopic disorders. | Differential methylation in three CpGs (*TSLP, GSTT1* and *CYB5R3*) between exposure groups, related to immune responses, cotinine metabolism and oxidative-reductive reaction respectively. Of these candidates, only *TSLP* validated by MDFS. *TSLP* 5’CGI methylation showed evidence for a difference between atopic dermatitis cases in the offspring. Also associated with downstream protein expression. |
| Xu et al, 2016 | 26384415 | 889 | Self-reported smoking during first trimester of pregnancy | 450K | Whole blood | Infancy | Re-analysis of data in PMID: 24906187 with Enmix method (models signal intensity with a flexible exponential-normal mixture distribution and truncated normal distribution to model background noise) and further preprocessing steps. Adjusted for cell type proportion, gestational age, sex, education, birth weight, maternal age, maternal BMI, parity, cleft phenotype and birth year. | 83 more CpG sites identified but biological role to not assessed. |
| Yang et al, 2015 | 26333704 | 9 subjects stratified into three groups: not exposed and no respiratory infection in infancy; exposed and respiratory infection; not exposed and respiratory infection | Mothers reported exposure to passive smoking and particulate matter | 450K | Cord blood | Birth | Investigated associations between pollutant exposure and DNA methylation, and between RTIs and DNA methylation. Adjusted for maternal age at delivery, maternal BMI, maternal education, gestational age, delivery mode, infant sex, family history of allergies. | Subjects with both RTIs and pollutant exposure differed from controls in 760 sites; subjects with RTIs but no pollutant exposure differed at 101 sites. Subjects with RTIs and pollutant exposure different from subjects with RTIs but no exposure in 4,550 sites. 15 CpG sites specifically associated with pollutant exposure. |

^1^ Technology: Technology used for epigenome-wide DNA methylation measurement where 450K refers to the Illumina Human Methylation 450 array and 27K refers to Illumina Human Methylation 27 array

Supplementary Table 2. Studies identified in literature review evaluating maternal micronutrient exposure and offspring epigenome-wide DNA methylation

| **Study** | **PubMed ID** | **Subjects (n)** | **Measure of exposure** | **Technology^1^** | **Tissue for methylation** | **Timepoint of offspring outcome** | **Methods** | **Key findings** |
| --- | --- | --- | --- | --- | --- | --- | --- | --- |
| Amarasekera et al., 2014 | 24891518 | 23 neonates recruited from a larger prospective birth cohort of mother-child pairs, separated into high folate (n=11) and low-folate (n=12) based on maternal serum folate levels. | Maternal serum folate from samples in the last trimester of pregnancy (≥28 weeks gestation) | 450K | 2 immune cell types (CD4^+^ and antigen-presenting cells) isolated from neonatal cord blood collected at birth | Birth | EWAS using principal components analysis to adjust for potential confounding factors. The first 15 principal components capturing >65% of the total variance were derived and tested for associations with clinical variables. | Reduced methylation related to maternal folate at a 923 bp region 3 kb upstream of the ZFP57 transcript |
| Binder et al., 2013 | 24305512 | 50 infants from a larger birth cohort study in Boston | Maternal RBC folate measured in samples taken at delivery | 27K | Placenta and cord blood | Birth | EWAS and mendelian randomization using two-stage least squares approach incorporating the MTHFR genotype. Adjustment for conception intention. | 7 CpGs were associated with maternal RBC folate levels, including genes involves in nucleic acid transport and metabolic processes. |
| Emes et al., 2012 | 23016625 | 18 cord blood samples from neonates (9 exposed to antiepileptic drugs; 9 unexposed) as well as high-dose folate supplementation before and during pregnancy (5 mg/day) | Maternal report of folate supplementation and AED use before/during pregnancy | 27K | Cord blood samples collected at delivery | Birth | Hierarchical clustering of CpGs of autosomal chromosomes (21,229 CpG sites) | AED associated with DNA methylation differences in offspring. Folate supplementation appeared to offset these differences. |
| Gonseth et al., 2015 | 26646725 | 343 mother-child pairs | Folate consumption 12 months prior to conception | 450K | Neonatal blood spots | Birth | EWAS of 319,264 CpGs passing quality control adjusting for covariates (gender, gestational age, cellular composition, principal components), and cell type in two independent sample sizes (n=167 and n=176). | Inverse association between preconception maternal folate levels and newborn DNA methylation across the genome. Top genes included LEF1, MAZ, TCF3. |
| Joubert et al., 2016 | 26861414 | 1,988 newborns from two European birth cohorts | Maternal plasma folate during pregnancy | 450K | Cord blood samples collected at delivery | Birth | Cohort-specific EWAS adjusting for covariates (maternal age, education, parity, maternal smoking) and cell type followed by fixed effects meta-analysis. 443 CpGs (320 genes) statistically significant with FDR correction; 48 with Bonferroni correction. | Most genes related to folate biology including APC2, GRM8, SLC16A12, OPCML, PRPH, LHX1, KLK4 and PRSS21. Other genes related to neural tube defects, neurological functions, and embryonic development. |
| Khulan et al. 2012 | 22307237 | 59 newborns of Gambian women enrolled in a placebo-controlled randomized trial for preconception micronutrient supplementation. | Micronutrient supplementation (treatment group) vs. placebo. | 27K | Newborn cord blood samples collected at delivery | Birth | Polynomial model using Illumina’s Genome Studio Methylation Module v1.8. | Nutritional supplementation corresponded to sex-specific differences in DNA methylation in cord blood and infant blood at age 9 months. Micronutrient supplementation impacted DNA methylation differences only in postnatal samples and not cord blood. |
| Mozhui et al, 2015 | 25742137 | 200 cord blood samples from African American (n=109) and European American participants (n=91) | Total folate level in plasma from mothers collected at 16-28 weeks of pregnancy | 27K | Cord blood collected at delivery | Birth | Methylation “M” values regressed on maternal folate with race, maternal age and estimated blood cell counts as covariates. | Folate had the strongest association with methylation of a CpG site within the WDR5 gene region. None of the CpG sites passed 5% FDR threshold. |

^1^ Technology: Technology used for epigenome-wide DNA methylation measurement where 450K refers to the Illumina Human Methylation 450 array and 27K refers to Illumina Human Methylation 27 array
